# Nephrology and experimental medicine *in vivo*, *in vitro*, and *in silico*

**DOI:** 10.1590/2175-8239-JBN-2024-0004en

**Published:** 2024-08-09

**Authors:** Mauricio Younes-Ibrahim

**Affiliations:** 1 Universidade do Estado do Rio de Janeiro, Rio de Janeiro, RJ, Brazil.; 2 Pontifícia Universidade Católica do Rio de Janeiro, Rio de Janeiro, RJ, Brazil.

**Keywords:** In vivo, *In vitr*o, In silico, Biomedical Research, In vivo, In vitro, In silico, Pesquisa Biomédica

## Abstract

Experimental medicine has formed the basis for generating medical knowledge for several centuries. The development of various experimental tools introduced at different times in medical practice has allowed the acquisition of knowledge with increasingly sophisticated scientific bases. Consequently, through *in vivo*, *in vitro* and, more recently, *in silico* experiments, we have witnessed an uninterrupted collection of experimental data potentially valuable for medicine, especially for Nephrology. We are gradually contemplating the uniqueness of individuals for the benefit of life and human dignity.

## Resumo

A medicina experimental tem formado a base para a geração de conhecimento médico ao longo de vários séculos. O desenvolvimento de diversas ferramentas experimentais introduzidas em diferentes momentos na prática médica tem permitido a aquisição de conhecimento com bases científicas cada vez mais sofisticadas. Em consequência, por meio de experimentos *in vivo*, *in vitro* e, mais recentemente, *in silico*, temos testemunhado uma coleta ininterrupta de dados experimentais potencialmente valiosos para a medicina, especialmente para a Nefrologia. Assim, contemplamos paulatinamente a singularidade dos indivíduos, em benefício da vida e da dignidade humana.

## Introduction

Although nephrology is a relatively young specialty, created only in the mid-20th century, it combines many activities that routinely involve the replacement of kidney function. By repeatedly resuscitating the Internal Environment, nephrology promotes a medical service that can extend the lives of (physiologically anephric) patients by days, months, years or even decades. As a highly complex specialty, nephrology incorporates a series of medical knowledge that has been acquired over the course of medical history, contributing to physiological, pathophysiological, and therapeutic understanding. Nephrological knowledge has particularly benefited from experimental medicine, building basic and applied scientific concepts that permeate nephrological practice from simple urine analysis to xenotransplantation.

## Discussion

**Figure 1:**
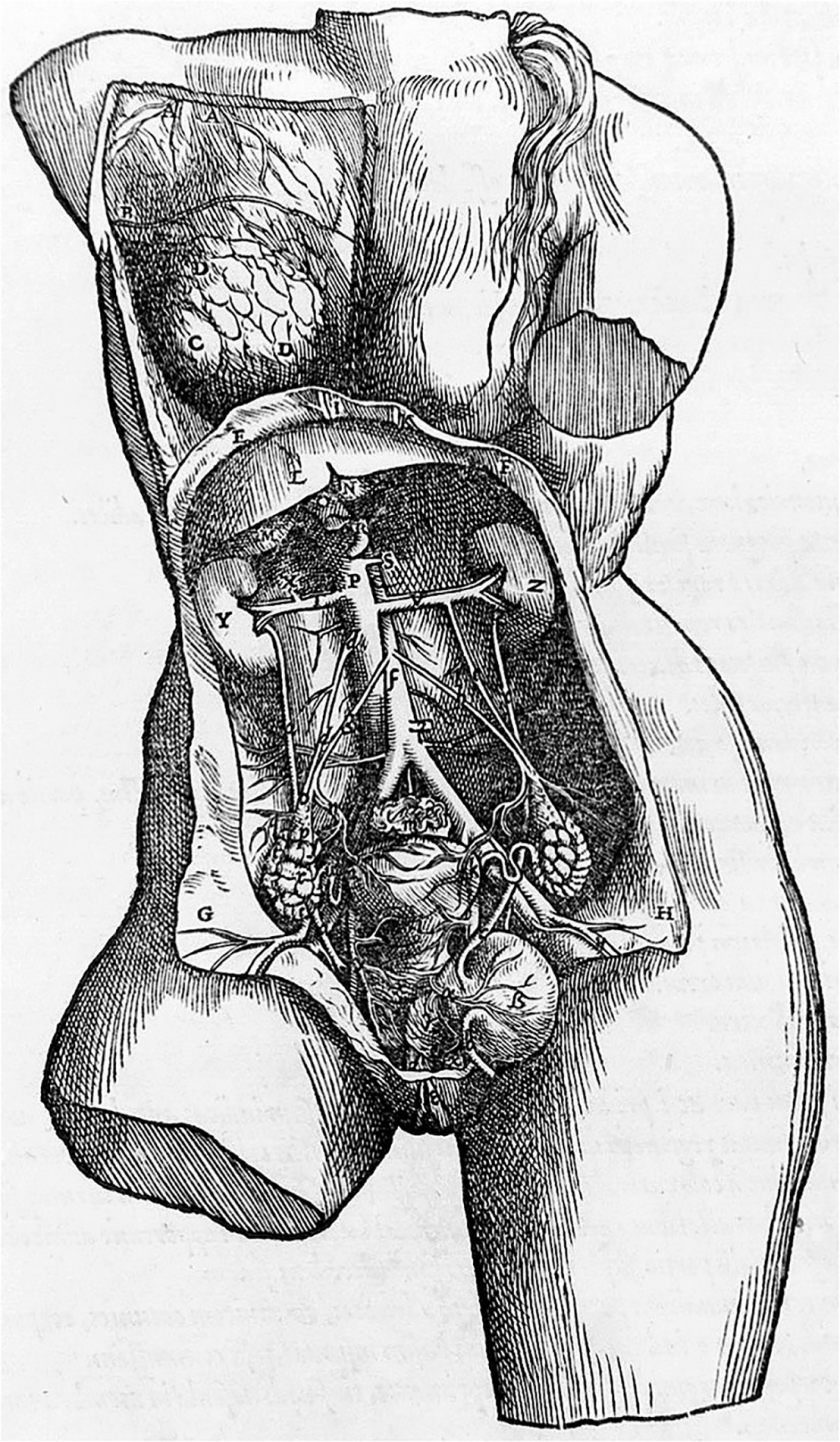
Drawing of the work of Vesalius (1543) that revolutionized knowledge and the teaching of anatomy in the 16th century^2^.

The 19th century, the “Century of Sciences”, was a period of systematization of thought and enthusiasm for discoveries. Until then, Western medical schools generated and transmitted descriptive knowledge supported by classical works (16th and 17th centuries) such as those of **Andreas Vesalius** ^1^, the “father of modern anatomy” (Figure 1), and **William Harvey** ^3^, the “father of physiology”. The knowledge of medicine was only visible macroscopically and *in vivo*. The insight of experienced observers represented the most effective source teaching the art of healing.

Microscopy was created in the 16th century when **Robert Hooke** ^4^ described the cell. **Marcello Malpighi** ^5^ (17th century) was the first anatomist to use it to identify erythrocytes and capillaries. In his work “De Viscerum Structura” from 1666 on human descriptive anatomy, Malpighi dedicates a section entitled “De Renibus”, in which he describes the structures of the kidney. In the 19th century, immersion techniques and binocular lenses contributed to histopathological studies and an in-depth knowledge of the cell nucleus. Medicine incorporated the visible *in vitro*, now on a microscopic scale.

In 1801, **Philippe Pinel** ^6^, the “father of modern psychiatry”, published the “Medical-philosophical Treatise on Mental Alienation”, in which he differentiates behavioral deviations from mental illness. Medicine, which until then had been limited to the clinic and surgery, inaugurated a new specialty with “Alienism”, which was humanistic and secular in its humanistic focus on the individual. Alienism brought patients together in hospitals to organize observation, classification, and treatment *in vivo*.

Also in the early 1800s, **Richard Bright** ^7^ began clinical studies and autopsies of patients with nephritis, characterizing “Bright’s Disease”. His pioneering work made him the “father of nephrology”.

The stethoscope, created in 1819 by **René Laennec** ^8^, established audible medicine, promoting semiological maneuvers and non-invasive *in vivo* experiments. Since the synthesis of urea (**Friedrich Wöhler** ^9^-1828), modern biochemistry emerged. **Friedrich Miescher** ^10^ described nucleic acids, **Louis Pasteur** ^11^ rejected “spontaneous generation”, and **Claude Bernard** ^12^ created the concepts of “Internal Environment” and gluconeogenesis.

While the theory of evolution (**Charles Darwin**-1859)^13^ caused controversy in academic circles, **Gregor Mendel** ^14^, the “father of genetics”, investigated heredity, innovating with the use of mathematical foundation of intergenerational macroscopy, published in 1865. In the same year, **Claude Bernard** introduced the binomial observation-experimentation in his “Introduction to the Study of Experimental Medicine”. Like **Descartes**, he aroused interest in the use of the scientific method, establishing a new path for medical-scientific thinking. Without knowing the intimacy of the cell and its molecular components, the “father of modern physiology” sought a hierarchical understanding in the biological symphony, heralding the dawn of renal physiology.

In the scientific boom of the 19th century, biological components, molecules, and chemical elements were gradually identified, supported by the periodic table recently created by **Dmitri Mendeleev** ^15^ (1869). Humanity was surprised to learn that the human body is not particular and is composed of the same matter found in other bodies in nature. During this period, there was a resurgence of rationalism, edited by **Hippocrates** (460 BC-377 BC), a thought that had lain dormant for centuries by theological currents applied to medicine. Biochemical processes and physical phenomena were gradually reproduced with the aid of test tubes. Medical thinking incorporated microscopy (microscopically visible medicine) and biochemistry (invisible medicine), inaugurating the promising era of cell biology.

**Table 1:** experimental medicine contributions to modern nephrology

Author	Century	Contribution	Ref.
Andrea Vesalius	XVI	Modern Anatomy	Vesalius^1^
William Harvey	XVII	Blood circulation	Harvey^3^
Robert Hooke	XVI	The Cell	Hooke^4^
Marcello Malpighi	XVII	Erythrocytes and Capillaries. The Kidney Structures	Motta^5^
Archibald Pitcairn	XVII	The Father of Mathematical Medicine	Ashrafian^26^
Richard Bright	XIX	Bright’s disease. The Father of Nephrology	Bright^7^
René Laënnec	XIX	Semiological Auscultation	Laënnec^8^
Friedrich Wöhler	XIX	Urea Synthesis	Wöhler^9^
Friedrich Miescher	XIX	Nucleic acids	Gorab and Leme^10^
Louis Pasteur	XIX	Microbiology	Debré and Forster^11^
Claude Bernard	XIX	Introduction to the Study of Experimental Medicine. The Father of Modern Physiology	Bernard^12^
Charles Darwin	XIX	Evolution theory	Darwin^13^
Gregor Mendel	XIX	The Father of Genetics	Mendel^14^
Dimitri Mendelejew	XIX	Periodic Table	Mendelejew^15^
Willian Osler	XIX	Principles and Practice of Medicine. The Father of Modern Medicine	Osler^16^
Wilhelm Röntgen	XIX	First X-ray	Röntgen^17^
Santiago Ramón y Cajal	XX	The Cajal cells. Nobel Prize in Medicine 1906	C. S. S.^18^
Elie Metchnikoff / Paul Ehrlich	XX	Immunology/Phagocytosis. Nobel Prize in Medicine 1908	Metchnikoff^19^
Willem Einthoven	XX	Electrocardiogram. Nobel Prize in Medicine 1924	Einthoven^20^
Willen Kolff	XX	Artificial Kidney. The Father of Artificial Organs	Kolff^21^ Wieringa et al.^22^
Peter Brian Medawar	XX	Immune Tolerance. The Father of Organ Transplants Nobel Prize in Physiology or Medicine 1960	Younes-Ibrahim^23^
James Watson/Francis Crick	XX	DNA Structure. Nobel Prize in Medicine 1962	Watson and Crick^24^
John Merrill/Joseph Murray	XX	First Kidney Transplant	Merrill et al.^25^
Archibald Pitcairn	XVII	The Father of Mathematical Medicine	Ashrafian^26^
Emmanuelle Charpentier/Jennifer Doudna	XXI	Crispr Method/DNA Editing Nobel Prize in Chemistry 2020	Derry^27^
Leonardo Riella	XXI	First Kidney Xenotransplant in Human	Rabin^28^

**Willian Osler** ^16^ combined clinic and pathology, identified platelets, and published “Principles and Practice of Medicine” (1892) being considered the “father of modern medicine.” Science emanated from *vivaria* and new research tools, with emphasis on spectroscopy. In 1895, **Wilhelm Roentgen** ^17^ surprised the world with the first x-ray (of his wife’s hand). He opened up a non-invasive way into the human body, inaugurating a new dimension of macroscopically “visible” medicine. The method found immediate application in the First World War. Non-invasive approaches have evolved in parallel with advances in physics. Ultrasonography and ultrasound, computed tomography, nuclear medicine, and nuclear magnetic resonance emerged. Visible medicine introduced functional imaging methods, enriching the tools of *in vivo* experimental medicine. At the same time, on a microscopic scale, **Santiago Ramón y Cajal** ^18^ inaugurated modern neuroscience by isolating brain nerve cells (Cajal cells) and was awarded the Nobel Prize in Physiology or Medicine (1906) alongside with **Camillo Golgi**.

**Edward Jenner**, the “father of immunology”, observed the protection of humans from smallpox by the cowpox virus in 1796. **Elie Metchnikoff** ^19^ (1882) conceptualized immunology, inaugurating the term phagocytosis and shared the Nobel Prize in Medicine with **Paul Ehrlich** in 1908.

Once the cells were known, the challenge was to cultivate them *in vitro*. The feat achieved with cell cultures at the beginning of the 20th century, boosted clinical and experimental medicine, from cell physiology to the development of organ and tissue transplants. Microscopically visible medicine was thus subjected to experimental conditions drawn from invisible medicine.

At the turn of the 20th century, **Willem Einthoven** ^20^ studied electrophysiology phenomena and was awarded the Nobel Prize in 1924 for the development of the electrocardiogram. Invisible aspects were graphically translated into electrophysiological information used clinically and experimentally *in vivo*.

During the Second World War, **Willen Kolff** ^21^ created the first blood bank in Europe in 1940, developed the prototype of an artificial kidney, and performed the first successful hemodialysis in 1945. Later, in 1967, **Kolff** ^22^ also developed the artificial heart and is considered the “father of artificial organs”.

In the 1950s, **Peter Brian Medawar** ^23^ developed the theory of Acquired Immune Tolerance, paving the way for the success of transplantation and received the Nobel Prize in Physiology or Medicine in 1960, being considered the “father of organ transplants”.

The nucleic acids described in 1869 revealed the structure of DNA in 1953 by **James Watson** and **Francis Crick** ^24^, the 1962 Nobel laureates. This marked the birth of molecular biology, which deals with the phenomena of replication, transcription, and translation involving DNA, RNA, and proteins. Sequential strategies made the genome project feasible, which was completed in 2003, involving 5000 researchers. Molecular biology provides information about the past, present, and future. Molecular panels and gene therapies provide therapeutic alternatives for genetic diseases and precision oncology. Through molecular biology, invisible medicine was finally incorporated into clinical practice and experimental medicine.

After decades of trials and *in vivo* and *in vitro* experiments, **John Merrill** and **Joseph Murray** ^25^ performed the first successful kidney transplant between identical twins in 1954. The development of immunosuppressive drugs allowed the first successful transplant from a deceased donor by the same team in 1962. These achievements led to **Murray** being awarded the Nobel Prize in Medicine in 1990.

In the 21st century, translational medicine emerged, a rapid connection from the bench to the bedside that aims to translate primary *in vitro* knowledge directly into clinical application *in vivo*.

Since **Archibald Pitcairne** ^26^, the “father of mathematical medicine” (17th century), the medical sciences no longer have to do without mathematics for biological axioms and the prediction of biological phenomena, automatically incorporating biostatistics and epidemiology. Mathematics is to 20^th^ and 21st century medical knowledge what the microscope was to the 19th century, especially in omics approaches (genomics, proteomics and metabolomics). The stages of knowledge were accelerated by the analytical scale and data processing of information technology. The “high-tech” tools herald the acquisition of innovative expertise, that flows visibly and invisibly into medical practice. In addition to numbers and letters, algorithms integrate biological models into artificial intelligence, bringing simulations and tests to the computer screen, sparing animals (*in vivo*), cells, or other biological elements (*in vitro*), inaugurating *in silico* experimental medicine.

Aided by information technology and the observation of the defense mechanisms of some bacteria that protect themselves from viruses, CRISPR (Clustered Regularly Interspaced Short Palindromic Repeats) was developed. This method made genome editing possible and represented a technological revolution by enabling the introduction of specific manipulations to modify the DNA of living cells, animals, or plants. **Emmanuelle Charpentier** and **Jennifer Doudna** ^27^ were awarded the 2020 Nobel Prize in Chemistry for this achievement. This technique changed the prognosis for a series of diseases caused by genetic disorders and allowed the therapeutic use of genetically modified cells and tissues.

Recently (March 2024), we have seen that the application of the CRISPR method in pig cells allowed the first xenotransplantation of a genetically modified pig kidney into a human patient, based on a series of previous experiments. This is a true milestone for clinical and experimental medicine. In this feat, the team led by **Leonardo Riella** ^28^ brought together a succession of multiple findings from experimental medicine *in vivo*, *in vitro*, and *in silico*.

In this 21st century, we are living in an auspicious new era, in which cybernetic tools are becoming increasingly important and triggering new waves of in silico testing. However, virtual medical reality must also be subordinated to the same elementary ethical principles of medicine and must never evade humanistic principles by preserving the ancient values that have governed the dignity of human life, in respect for the “medicine of the soul” since the beginning of time.

## Conclusion

Modern medicine is evolving into precision medicine, focusing on prevention and the uniqueness of the individual rather than just the general characteristics of diseases. The various stages that medical knowledge has passed through in the course of history have not been without experimental medicine, whose modern tools make it possible to be practiced *in vivo*, *in vitro*, and *in silico*, for the benefit of life and human dignity.
